# Prostate cancer presentation and management in the Middle East

**DOI:** 10.1186/s12894-024-01427-6

**Published:** 2024-02-09

**Authors:** Mutlay Sayan, Astrid Langoe, Ozlem Aynaci, Ayfer Ay Eren, Mehmet Fuat Eren, Ilke Onur Kazaz, Zainab Ibrahim, Omar Tama Al-Akelie, Loma Al-Mansouri, Ramiz Abu-Hijlih, Shalini Moningi, Elia Abou Chawareb, Albert El Hajj, Peter F. Orio, Layth Mula-Hussain

**Affiliations:** 1 Brigham and Women’s Hospital and Dana-Farber Cancer Institute, Harvard Medical School, Boston, MA USA; 2 Boston College, Boston, MA USA; 3 Karadeniz Technical University, Trabzon, Türkiye Turkey; 4 Kartal Dr. Lütfi Kirdar Education and Research Hospital, Istanbul, Turkey; 5 Marmara University, Istanbul Pendik Education and Research Hospital, Istanbul, Türkiye Turkey; 6 King Hamad University Hospital, Al Sayh, Bahrain; 7 Alamal National Hospital for Cancer Management, Baghdad, Iraq; 8 Basrah Medical College, Basra, Iraq; 9 King Hussein Cancer Center, Amman, Jordan; 10 American University of Beirut Medical Center, Beirut, Lebanon; 11 Faculty of Medicine, Dalhousie University, Halifax, NS Canada; 12 College of Medicine, Ninevah University, Mosul, Ninevah Iraq; 13 Department of Radiation Oncology, Dana-Farber Cancer Institute, Brigham and Women’s Hospital, Harvard Medical School, 75 Francis Street, Boston, MA 02115 USA

**Keywords:** Prostatic neoplasms, Middle East, Disease presentation, Disease management, Diagnostic challenges

## Abstract

**Background:**

Although prostate cancer is a prevalent malignancy worldwide, its clinical presentation and management in the Middle East are not well-documented. This study aims to provide insights into the initial clinical presentation and management of prostate cancer in this region.

**Methods:**

A retrospective review was conducted on seven institutional databases from six Middle Eastern countries, including Türkiye, Lebanon, Iraq, Syria, Bahrain, and Jordan, to identify patients diagnosed with prostate cancer in 2021. Descriptive analysis was performed on the collected data to provide an overview of the demographic, clinical, and treatment variables.

**Results:**

A total of 1,136 patients were identified with a median age of 70 (range, 50–84). Most patients (78%) received their prostate cancer diagnosis after presenting with symptoms, as opposed to routine PSA screening. At the time of diagnosis, 35% of men had clinical T3 or T4 disease, 54% with Stage IV disease and 50% with Gleason score ≥ 8. Regarding treatment, 20% of non-metastatic and 22% of metastatic patients received no treatment.

**Conclusion:**

Most men in this study sought prostate cancer evaluation due to symptoms and were subsequently diagnosed with advanced-stage disease, providing a foundation for future research aimed at understanding the underlying factors behind the observed trends and enabling informed interventions.

## Background

Prostate cancer is one of the most common types of cancer affecting men worldwide [1]. Its complex nature presents a significant health burden that varies across geographic regions, age groups, and racial/ethnic populations [2]. Genetic predisposition, lifestyle, and healthcare access are important to consider in the diagnosis and prognosis of this disease [3, 4]. While there have been substantial advancements in understanding etiology and treatment strategies, prostate cancer still remains a leading cause of cancer-related morbidity and mortality among men globally.

In contrast to regions like Europe and North America, where extensive research on prostate cancer has been conducted, the Middle East presents a different scenario. The status of prostate cancer in this region remains notably underexplored, resulting in a fragmented understanding of its initial presentation and management [5–7]. One significant reason for this disparity is the absence of well-established cancer registries. However, limited available data suggests a consistent rise in the incidence of prostate cancer over the past decade [8]. This increasing incidence highlights the pressing need for comprehensive research efforts in the Middle East. Grasping the disease stage at the initial presentation is a pivotal first step in this direction. Such insights not only provide a clearer picture of the current state of prostate cancer care in the region but also provide the essential data to craft and implement effective policies.

Despite the global prevalence of prostate cancer, there remains a significant knowledge gap concerning its manifestation and management in the Middle East. This study aims to comprehensively assess prostate cancer’s clinical presentation and management in the region, providing a foundation for future research and informed policy-making.

## Materials and methods

A retrospective review was conducted on institutional databases from six Middle Eastern countries, Türkiye, Lebanon, Iraq, Syria, Bahrain, and Jordan, to identify patients diagnosed with prostate cancer in 2021. A comprehensive review of seven databases from various institutions was conducted, encompassing six public and one private health care system, all of which are affiliated with academic centers. Prostate cancer diagnosis was confirmed by the treating physician at each participating institution following pathological confirmation. Tissue samples for histopathological analysis were obtained through various methods, including prostate biopsy, biopsy of metastatic sites, or from tissue acquired during radical prostatectomy. Data were collected on demographic details, clinical presentations, and treatment modalities. The staging of prostate cancer was performed by combining clinical assessment, radiological imaging, and pathological findings, following the guidelines outlined in the American Joint Commission on Cancer staging manual, 8th edition. Descriptive analysis was performed on the collected data to provide an overview of the demographic, clinical, and treatment variables.

## Results

A total of 1,136 patients were identified with a median age of 70 (range, 50–84). The baseline patient characteristics are shown in Table 1. Most patients (78%) were diagnosed after developing symptoms and not on routine PSA screening. Diagnostic workup was completed in 87% of the patients. At the time of diagnosis, 35% of men presented with clinical T3 or T4 disease, 54% with Stage IV disease and 50% with Gleason score ≥ 8. The mean PSA at the time of presentation was 84 ng/ml.

**Table 1:** Demographic and clinical characteristics of the study cohort

Age, median, range	70 (50–84)
**Reason for prostate cancer evaluation, No. (%)**	
Routine surveillance	254 (22)
Patient had symptoms	882 (78)
**AJCC tumor stage, No. (%)**	
T1c	90 (10)
T2a	110 (12)
T2b	166 (18)
T2c	235 (25)
T3a	148 (16)
T3b	90 (10)
T4	86 (9)
**AJCC nodal stage, No. (%)**	
N0	556 (64)
N1	311 (36)
**AJCC metastatic stage, No. (%)**	
M0	587 (52)
M1a	30 (3)
M1b	268 (24)
M1c	251 (22)
**AJCC stage**	
Stage I	86 (7)
Stage II	218 (19)
Stage III	223 (20)
Stage IV	609 (54)
**Gleason score, No. (%)**	
6 (3 + 3)	106 (11)
7 (3 + 4)	169 (18)
7 (4 + 3)	191 (20)
8 (4 + 4, 3 + 5, or 5 + 3)	225 (24)
9 or 10 (4 + 5, 5 + 4, or 5 + 5)	246 (26)
**Baseline PSA, mean**	83.4
**Diagnostic Imaging, No. (%)**	
Bone scan*	4 (0)
CT scan*	42 (4)
MRI*	148 (13)
PSMA-PET, with or without other imaging	372 (33)
US alone*	54 (5)
Bone scan + CT scan	93 (8)
Bone Scan + CT scan + MRI	272 (24)
No imaging	151 (13)

*Abbreviations* AJCC, American Joint Committee on Cancer; PSA, prostate-specific antigen; CT, computed tomography; MRI, magnetic resonance imaging; PSMA-PET, prostate-specific membrane antigen positron emission tomography; US, ultrasound

*Only the respective imaging modality was used without combining with others

Treatment details are shown in Table 2. Among the non-metastatic patients, 24% underwent a radical prostatectomy, 48% received definitive radiotherapy with or without androgen deprivation therapy (ADT), 8% received ADT alone, and 20% received no treatment. No patients received brachytherapy. Hypofractionated radiotherapy was used in 49% of patients. Among the metastatic patients, 56% received ADT with or without additional systemic therapy, 22% had palliative radiotherapy, and 22% received no treatment.

**Table 2:** Treatment characteristics of the study cohort

**Treatment for non-metastatic patients, No. (%)**	
No treatment	106 (20)
Radical prostatectomy alone	84 (16)
Radiotherapy alone	28 (5)
ADT alone	45 (8)
Radical prostatectomy + radiotherapy + ADT	30 (6)
Radical prostatectomy + ADT	10 (2)
Radiotherapy + ADT	228 (43)
**Treatment for metastatic patients, No. (%)**	
No treatment	132 (22)
ADT +/- additional systemic therapy	343 (56)
Palliative radiotherapy to metastases	140 (22)

*Abbreviations* ADT, androgen deprivation therapy

In this study cohort, the distribution of patients with locally advanced disease was as follows: 66 patients (5%) had regional and local lymph node involvement alone (N1 disease), with the majority undergoing definitive radiation therapy in combination with ADT (67%). Additionally, a subset of 20 patients (2%) presented with clinical T3-T4 disease alone, without evidence of lymph node involvement or distant metastasis, with the majority of these patients undergoing radical prostatectomy, either with or without ADT (80%).

## Discussion

In this study, we conducted a comprehensive retrospective review of 1,136 patients across seven institutional databases in six Middle Eastern countries to understand prostate cancer’s clinical presentation and management strategies in the region. Our findings indicate most patients were diagnosed after developing symptoms rather than through routine PSA screenings, with a notable number presenting with advanced-stage disease (54% with Stage IV disease) and not receiving any form of treatment (22%). The clinical significance of this study lies in its potential to serve as a foundation for designing future studies aimed at better understanding the underlying reasons behind these findings, ultimately enabling the development of informed interventions.

Recent studies have highlighted the importance of age and serum PSA levels as major risk factors for prostate cancer. A comprehensive multicenter case study revealed that the estimated odds ratios for prostate cancer increase significantly in different age groups corresponding to PSA levels. Specifically, for individuals in their forties with PSA levels between 2.6 and 4 ng/ml, the risk is approximately 12.5 times higher [9]. Moreover, recent findings from a randomized control trial demonstrate the efficacy of PSA screening in reducing metastasis and mortality in men aged 55–74 years. After a median follow-up of 21 years, the study reports rate ratios of prostate cancer-specific mortality at 0.73 (95% CI: 0.61–0.88) and metastasis at 0.67 (95% CI: 0.58–0.78), highlighting the benefits of screening [10].

Several points require further discussion. First, the retrospective design inherently introduces confounding factors that cannot be entirely accounted for. Therefore, these results are hypothesis-generating and should be evaluated in a new cohort study. Despite this limitation, our findings align with the high rates of advanced-stage disease presentation observed in small institutional databases [11, 12]. One major contributing factor to this observation could be the lack of prostate cancer awareness, as a significant proportion of patients (78%) in this study sought prostate cancer evaluation due to symptoms, indicating that many may not have been aware of the need for routine screening. Another contributing factor could be the limited practice of routine prostate cancer screening in the region by physicians. Multiple barriers may contribute to this, including a lack of healthcare professional training and knowledge, limited resources or access to screening equipment, time constraints during medical consultations, or the fact that prostate cancer screening may be considered a lower priority compared to other pressing health concerns. To the best of our knowledge, there is currently no comprehensive study assessing the level of prostate cancer awareness or the status of prostate cancer screening practices in the Middle East. Investigating this area would provide valuable insights into the barriers and challenges faced by healthcare systems and individuals in accessing timely prostate cancer diagnosis, thereby contributing to a more comprehensive understanding of the issue. Furthermore, the limited availability of advanced imaging modalities, such as PSMA PET scans [13] and multiparametric-MRI [14], in this region may have contributed to the presentation of prostate cancer at more advanced stages.

Second, while 20% of patients with non-metastatic disease did not receive any treatment, it’s worth noting that active surveillance, a management strategy often used for low-risk prostate cancer, might have been chosen for these patients. In contrast, a similarly high rate of no treatment (22%) was observed among patients with metastatic disease, in which cases active surveillance is not typically a preferred option. ADT stands as the cornerstone of treatment for metastatic prostate cancer. Furthermore, numerous randomized controlled trials have demonstrated that combination therapy, which incorporates ADT alongside androgen receptor-signaling inhibitors or docetaxel, significantly improves overall survival compared to ADT alone [15–19]. Additionally, the efficacy of radiotherapy in the context of metastatic prostate cancer has been well-established through multiple prospective studies [20–23]. Given the compelling evidence supporting the treatment of patients with metastatic prostate cancer, it remains unclear why 22% of patients with metastasis in this cohort did not receive any form of treatment. This discrepancy raises important questions about the factors contributing to the omission of treatment, which warrants further investigation.

Third, the emergence of the COVID-19 pandemic brought forth unique challenges in healthcare delivery. Notably, our study did not consider the specific impact of COVID-19 on cancer diagnosis and treatment rates. Consequently, the question remains whether the proportion of patients presenting with advanced-stage disease or not receiving treatment would have been similar in the absence of the pandemic [24].

In North America and Europe, prostate cancer is typically detected through PSA testing for early diagnosis, resulting in a significant rise in disease incidence. Consequently, prostate cancer is the most commonly diagnosed cancer among men in these regions [25]. The majority of cases are identified while the cancer remains localized within the prostate, leading to a 5-year survival rate of approximately 98% [25]. However, the Middle East presents a distinct scenario, as evidenced by our study, where the majority of patients (78%) receive their prostate cancer diagnosis after developing symptoms, and 54% of men have Stage IV disease at diagnosis. This indicates a later stage of detection compared to North America and Europe. In Africa, prostate cancer is characterized by higher PSA values, a more frequent presentation of locally advanced cancer, and high-grade disease [26]. This suggests that the disease may be more advanced at the time of diagnosis in this region, similar to the situation in the Middle East.

## Conclusion

In this comprehensive retrospective review of prostate cancer cases across six Middle Eastern countries revealed a concerning pattern where most men sought prostate cancer evaluation due to symptoms and were subsequently diagnosed with advanced-stage disease, while a substantial portion received no treatment. These data should guide future studies to understand the underlying reasons behind these trends, ultimately enabling well-informed interventions to improve prostate cancer care and outcomes in the region.

## Data Availability

No datasets were generated or analysed during the current study.
